# Supplementary material for: Topographic generation of submesoscale centrifugal instability and energy dissipation
Source: Nat Commun. 2016 Sep 29;7:12811. doi: 10.1038/ncomms12811 (PMC5056419; doi:10.1038/ncomms12811)
Supplement: Supplementary Information — Supplementary Figures 1-4 and Supplementary Methods [file ncomms12811-s1.pdf]

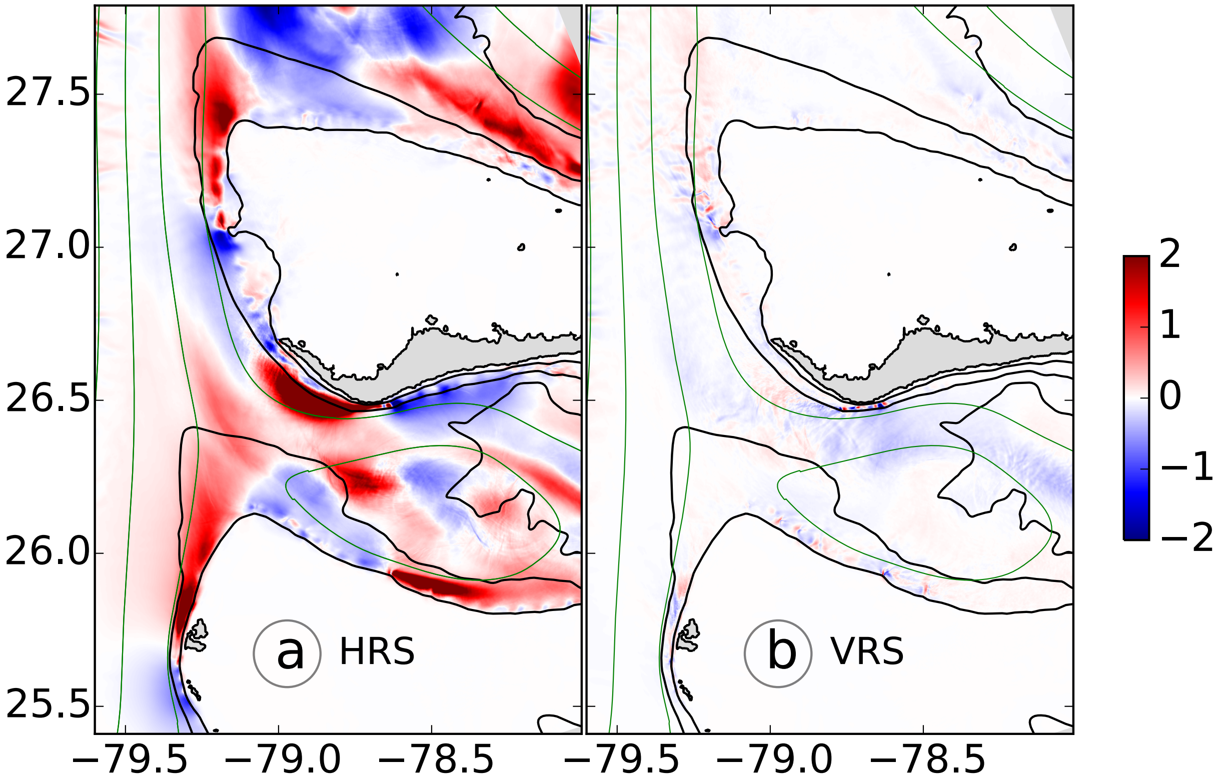

Supplementary Figure 1: **Identification of centrifugal instability**. Comparison between (a) the horizontal shear production term HRS $= -\overline{\mathbf{u}'v'} \cdot \frac{\partial \overline{\mathbf{u}}}{\partial y} - \overline{\mathbf{u}'u'} \cdot \frac{\partial \overline{\mathbf{u}}}{\partial x}$ and (b) the vertical shear production term VRS = $-\overline{\mathbf{u}'w'} \cdot \frac{\partial \overline{\mathbf{u}}}{\partial z}$. The mean transport streamfunction is shown in green with a 5 Sv interval (1 Sv = 10^6 m^{-3}s^{-1}). Units for energy rates are W m kg^{-1}. Topography is shown in black contours at 0 m, 100 m, 500 m, and 1000 m isobaths.

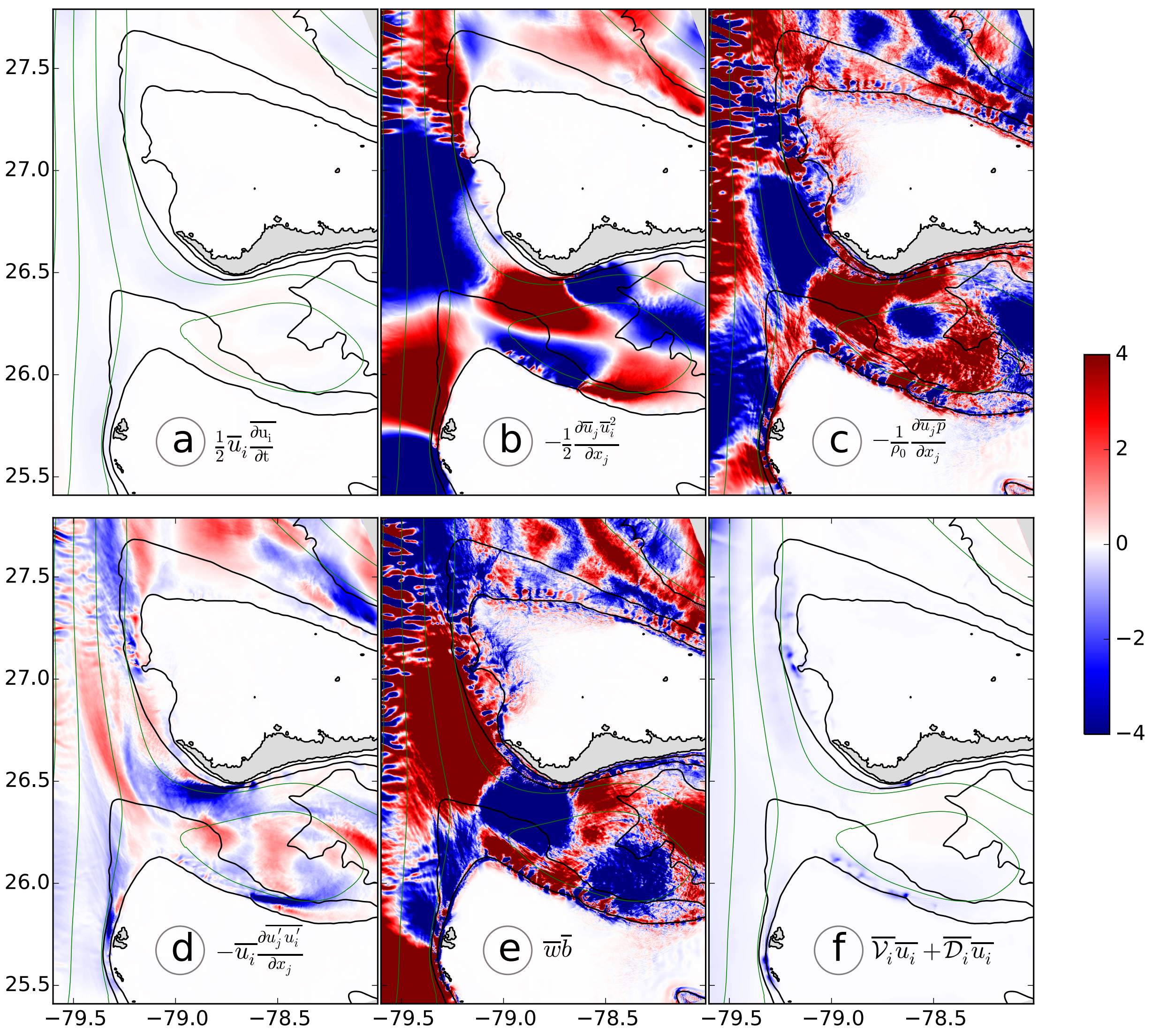

Supplementary Figure 2: **Mean kinetic energy balance**. Time mean depth integrated terms of the mean kinetic energy (MKE) equation (Equ. 1). The mean transport streamfunction is shown in green with a 5 Sv interval (1 Sv = 10^6 m^{-3}s^{-1}). Units for energy rates are W m kg^{-1}. Topography is shown in black contours at 0 m, 100 m, 500 m, and 1000 m isobaths.

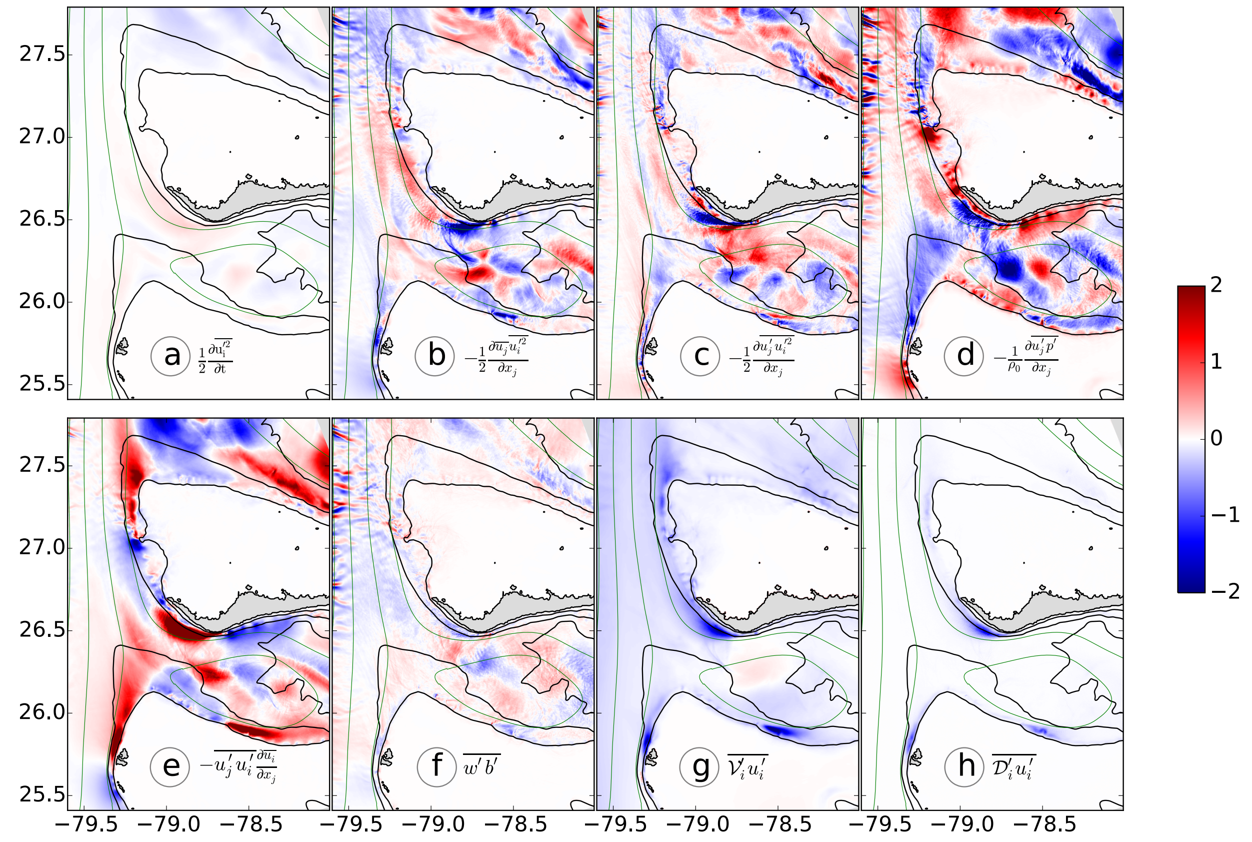

Supplementary Figure 3: **Eddy kinetic energy balance**. Time mean depth integrated terms of the eddy kinetic energy (EKE) equation (Equ. 2). The mean transport streamfunction is shown in green with a 5 Sv interval (1 Sv = 10^6 m^{-3}s^{-1}). Units for energy rates are W m kg^{-1}. Topography is shown in black contours at 0 m, 100 m, 500 m, and 1000 m isobaths.

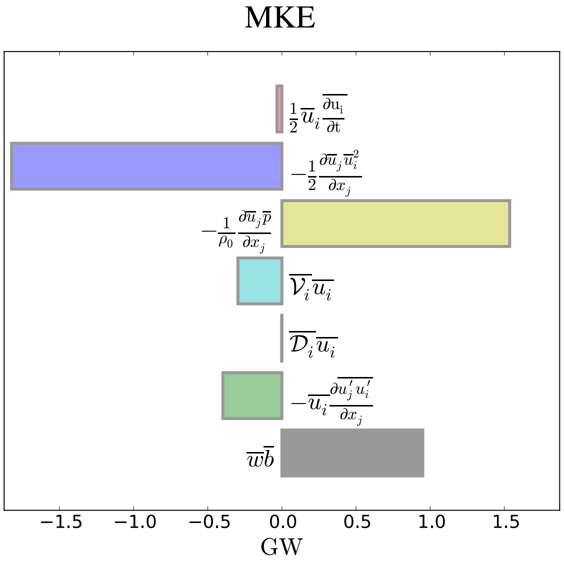

Supplementary Figure 4: **Mean kinetic energy budget**. Volume integral of the mean kinetic energy balance equation terms over the domain plotted in Figs. 2 and 3. Units for energy rates are GW (10^9 W).

Supplementary Methods

Energy Balance. The mean kinetic energy of the flow $\mathrm{KE} = \frac{1}{2}(\overline{u^2} + \overline{v^2})$ is the sum of the kinetic energy of the mean flow, $\mathrm{MKE} = \frac{1}{2}(\overline{u}^2 + \overline{v}^2)$, and the eddy kinetic energy, $\mathrm{EKE} = \frac{1}{2}(\overline{u'^2} + \overline{v'^2})$, where the overbar denotes a time average, and the prime denotes fluctuations relative to the time average.

The mean kinetic energy equation is formed by taking the inner product of the mean horizontal velocities with the mean terms in the momentum equations,:

$$\frac{1}{2}\overline{u}_i\frac{\partial\overline{\mathrm{u_i}}}{\partial\mathrm{t}} + \underbrace{\frac{\partial\left(\frac{1}{2}\overline{u}_j\overline{u}_i^2 + \frac{1}{\rho_0}\overline{u}_j\overline{p}\right)}{\partial x_j}}_{\text{Boundary Transport}} = \underbrace{-\overline{u_i}\frac{\partial\overline{u_j'u_i'}}{\partial x_j}}_{\text{EKE}\to\text{MKE}} + \underbrace{\overline{w}\overline{b}}_{\text{MPE}\to\text{MKE}} + \underbrace{\overline{\mathcal{V}_i}\overline{u_i}}_{\text{Vertical mixing}} + \underbrace{\overline{\mathcal{D}_i}\overline{u_i}}_{\text{Horizontal diffusion}} \quad , \quad (1)$$

where Cartesian tensor notation with summation convention has been used, $i = 1, 2$, $j = 1, 2, 3$; u_i are the horizontal components of the velocity vector u_j; $u_3 = w$ is the vertical velocity; p is the pressure anomaly; $b = -\frac{g\rho}{\rho_0}$ is the buoyancy anomaly; $\mathcal{V}_i$ and $\mathcal{D}_i$ are the vertical mixing and horizontal diffusion terms in the horizontal momentum equations.

The eddy kinetic energy equation is formed by subtracting the energy equation of the mean flow from that of the total flow:

$$\frac{1}{2}\frac{\partial\overline{\mathrm{u_i'^2}}}{\partial\mathrm{t}} + \underbrace{\frac{\partial\left(\frac{1}{2}\overline{u_j}\,\overline{u_i'^2} + \frac{1}{2}\overline{u_j'u_i'^2} + \frac{1}{\rho_0}\overline{u_j'p'}\right)}{\partial x_j}}_{\text{Boundary Transport}} =$$

$$\underbrace{-\overline{u_j'u_i'}\frac{\partial\overline{u_i}}{\partial x_j}}_{\text{MKE}\to\text{EKE}} + \underbrace{\overline{w'b'}}_{\text{EPE}\to\text{EKE}} + \underbrace{\overline{\mathcal{V}_i'u_i'}}_{\text{Vertical mixing}} + \underbrace{\overline{\mathcal{D}_i'u_i'}}_{\text{Horizontal diffusion}} \quad . \quad (2)$$
